# Exogenous Hydrogen Sulfide Offers Neuroprotection on Intracerebral Hemorrhage Injury Through Modulating Endogenous H_2_S Metabolism in Mice

**DOI:** 10.3389/fncel.2019.00349

**Published:** 2019-08-07

**Authors:** Haiyan Shan, Jianping Qiu, Pan Chang, Yang Chu, Cheng Gao, Haocheng Wang, Guang Chen, Chengliang Luo, Tao Wang, Xiping Chen, Mingyang Zhang, Luyang Tao

**Affiliations:** ^1^Institute of Forensic Sciences, Soochow University, Suzhou, China; ^2^Department of Obstetrics and Gynecology, The Affiliated Suzhou Hospital of Nanjing Medical University, Suzhou, China; ^3^Central Laboratory, The Second Affiliated Hospital of Xi’an Medical College, Xi’an, China; ^4^School of Pharmacy, Soochow University, Suzhou, China

**Keywords:** hydrogen sulfide, intracerebral hemorrhage, autophagic cell death, apoptosis, mice

## Abstract

Hydrogen sulfide (H_2_S), an important endogenous signaling molecule, has a significant neuroprotective role in the central nervous system. In this study, we examined the protective effects of exogenous H_2_S against intracerebral hemorrhage (ICH), as well as its underlying mechanisms. We investigated the effects of exogenous H_2_S on ICH using Western blotting, injury volume, measurement of brain edema, propidium iodide (PI) staining, and behavior assessment, respectively. We found that endogenous H_2_S production was downregulated in the brain after ICH, which is caused by the decrease in cystathionine β-synthase (CBS) as the predominant cerebral H_2_S-generating enzyme in the brain. Treatment with sodium hydrosulfide (NaHS; an H_2_S producer) could restore the H_2_S production and the expression of CBS. NaHS could also attenuate brain edema, injury volume, and neurological deficits in the Morris water maze test after ICH. Western blotting results indicated that H_2_S pretreatment reversed the increase in caspase 3 cleavage and the decrease in Bcl-2, suppressed the activation of autophagy marker (LC3II and Beclin-1), and maintained the p62 level in injured striatum post-ICH. However, H_2_S could not restore brain CBS expression and H_2_S content, reduce brain edema, and improve motor performance and memory function after ICH through modulating autophagy and apoptosis when pretreated with the CBS inhibitor aminooxyacetic acid (AOAA). We also found that AOAA reduced the endogenous H_2_S production through inhibiting the enzyme activity of CBS rather than modulating the expression of CBS protein level. These present results indicate that H_2_S may possess potential therapeutic value in the treatment of brain injury after ICH, and the protective effect of exogenous H_2_S against ICH may be not a direct action but an indirect effect through inducing endogenous H_2_S metabolism responses.

## Introduction

Intracerebral hemorrhage (ICH) is the second most common subtype of stroke (up to 15% of all strokes) leading to high mortality and morbidity throughout the world (van Asch et al., 2010; Kim and Bae, 2017). The post-ICH brain injury can be distinguished into primary brain injury caused by disruption and mechanical deformation of brain tissue due to hematoma growth, and secondary brain injury induced by microglia activation, mitochondrial dysfunction, and neurotransmitter and inflammatory mediator release, which lead to cell death, including apoptosis and autophagy (Bobinger et al., 2018). These cell death pathways lead to the removal of inactivated and damaged cells and also result in neuronal cell damage and poor neurological outcome (Lin et al., 2018). Recent evidence indicates that compensatory response as a strategy offers promising opportunities for each participant to improve brain function following brain injury (Tovar-y-Romo et al., 2016; Rodriguez et al., 2017). To develop a suitable neuroprotective agent, which can enhance the protective pathways to treat hemorrhage stroke, may be regarded as a promising treatment option when transitioned to the clinical setting.

Hydrogen sulfide (H_2_S) is most recently found to be a novel gasotransmitter signaling molecule, which can modulate cellular biological function related to health and diseases (Li et al., 2011). The effects of H_2_S have been extensively researched, including the regulation of inflammation, cell death, and cellular metabolism (Gopalakrishnan et al., 2018). Recent basic medical studies and preclinical studies on neurological diseases have demonstrated that treatment with H_2_S at physiological or pharmacological levels attenuates brain injury (Zhang et al., 2013; Zhang J.Y. et al., 2017). Zhao et al. reported that H_2_S attenuated NLRP3 inflammasome-mediated neuroinflammation after ICH (Zhao et al., 2017). They also found that supplement with taurine could increase H_2_S content, enhanced cystathionine β-synthase (CBS) expression, and effectively mitigated the severity of pathological inflammation after ICH (Zhao et al., 2018). Despite the performance of the protective role of H_2_S in neuroinflammation after ICH, how H_2_S regulates cell death including apoptosis and autophagy in ICH remains unclear. Our previous study demonstrated that H_2_S provided neuroprotection in traumatic brain injury (TBI) via inhibition of autophagy and apoptosis (Zhang et al., 2014). We also found that ICH induced the activation of autophagy, and inhibition of autophagy was recognized as a therapeutic strategy of brain injury after hemorrhage in an ICH model (Shen et al., 2016). Based on the findings mentioned above, a mouse model of ICH was established to evaluate therapeutic efficacy and neuroprotective mechanism of H_2_S against brain injury.

Obviously, H_2_S is generated from cysteine by CBS in the brain and regulates different cellular physiological functions such as ion channel and release of neurotransmitters by acting as intracellular signaling molecules (Kamat et al., 2015). Neurological effects of H_2_S recently were put forward to account for the regulation of circulating sulfide rather than endogenous production (Olson et al., 2008). This was disproved based on recent studies showing that H_2_S found in the CNS is more likely to be derived directly from the brain than from the blood (Whiteman et al., 2004; Kim et al., 2011). Moreover, no clear mechanisms of exogenous H_2_S modulating endogenous H_2_S pathway were addressed, and biological effects of endogenous H_2_S synthase inhibitors were not investigated after ICH. In order to elucidate the molecular mechanism of exogenous H_2_S, we also added the endogenous H_2_S synthase inhibitor to investigate whether exogenous H_2_S exerts a protective effect against brain injury through endogenous H_2_S pathway.

## Materials and Methods

### Animals and Drug Treatments

Adult male CD1 mice with an average body weight of 23 g (20–25 g) were used in this study. Sodium hydrosulfide (NaHS), an H_2_S donor, was obtained from Sigma-Aldrich (St. Louis, MO, United States) and dissolved in saline. *O*-(carboxymethyl) hydroxylamine hemihydrochloride (AOAA), an H_2_S inhibitor, was obtained from Sigma-Aldrich (St. Louis, MO) and dissolved in saline. For drug time effects assays, NaHS was intraperitoneally (i.p.) injected 30 min before or 30 min, 1, 2, 4, or 6 h after ICH. For drug dosage effects assays, NaHS (1, 10, 25, 50, and 100 μmol/kg) was i.p. injected 30 min before ICH. AOAA was i.p. injected 1 h before ICH. Animals were grouped into sham, ICH, ICH + NaHS, ICH + AOAA, and ICH + NaHS + AOAA groups. Sham-injured mice received craniotomy and injection with saline without ICH. All the animal procedures were approved by the Institutional Animal Use and Care Committee at Soochow University and conducted in accordance with the guidelines of animal use and care of the National Institutes of Health (NIH) and the ARRIVE (Animal Research: Reporting *In Vivo* Experiments). All efforts were made to minimize the numbers of animals used and ensure minimal suffering. In all experiments, data were obtained by investigators blinded to study group.

### ICH Model

The ICH model was used as previously described (Wang et al., 2013). The CD1 mice were deeply anesthetized with chloral hydrate (4% solution), and surgery was performed under aseptic conditions and mounted in a stereotaxic system (David Kopf Instruments, Tujunga, CA, United States). The following steps were all performed using aseptic techniques. A midline incision on the scalp exposed the skull without requiring muscle retraction. Craniotomy was performed by handheld trephine and injected with Type IV collagenase (0.075 U in 500 nl of saline) unilaterally into the left striatum at the following stereotactic coordinates: 1 mm anterior and 2.0 mm lateral of the bregma, 3.5 mm in depth. Collagenase was delivered over 5 min. The needle stayed in place for an additional 5 min to prevent reflux. The craniotomy was sealed with bone wax, and the scalp was sutured. Mice in the sham group were only subjected to sterile saline injection. The overall mortality rate was <2%. All mice were allowed to fully recover under observation. Body temperature was maintained at 36.5–37.5°C by means of a heating blanket and a lamp throughout the procedure from the start of the surgery until the animals recovered from anesthesia. Animals were housed under a 12-h light/dark cycle in a pathogen-free area with free access to water and food. All surgical interventions and post-operative animal care were carried out in accordance with the NIH *Guide for the Care and Use of Laboratory Animals* and were approved by the Chinese National Committee to the Use of Experimental Animals for Medical Purposes, Jiangsu Branch. All efforts were made to minimize the number of animals used and their suffering.

### Cerebral Water Content (CWC) Measurements

For time course of ICH-induced brain edema evaluation, animals were anesthetized with 4% chloral hydrate and decapitated at 1 h, 6 h, 12 h, 1 day, 2 days, 3 days, and 7 days after ICH. For drug dosage effect and time validity evaluation, animals were anesthetized with 4% chloral hydrate and decapitated 24 h after ICH. The brains were removed and placed in a glass petri dish. CWC was measured with a drying method (Shohami et al., 1993). The cerebellar tissue was discarded, the right and left hemispheres were separated along the anatomic midline, and the wet weight of each hemisphere was measured. The tissues were completely dried in an oven at 100°C for 5 days, and the dry weight of each hemisphere was recorded. The percentage water content (% water) was calculated according to the Elliott formula for each hemisphere: %water = [(wet weight – dry weight)/wet weight] × 100 (Bierbach et al., 2008).

### Evaluation of Motor and Morris Water Maze (MWM) Performance

Vestibulomotor function was assessed using a wire grip test (Bermpohl et al., 2006). Mice were placed on a metal wire (45 cm long) suspended 45 cm above a foam pad and were allowed to traverse the wire for 60 s. The latency that a mouse remained on the wire within a 60-s interval was measured, and wire grip scores were quantitated using a 5-point scale. A score of 1 point was given if the mouse failed to hold on to the wire with both sets of forepaws and hind paws together; 2 points were given if the mice held on to the wire with both forepaws and hind paws but not the tail; 3 points were given if the mouse used its tail along with both forepaws and both hind paws; 4 points were given if the mouse moved along the wire on all four paws plus tail; and 5 points were given if mice that scored 4 points also ambulated down one of the posts used to support the wire. Mice that were unable to remain on the wire for less than 30 s were given a score of zero. The wire grip test was performed in triplicate, and an average value was calculated for each mouse on each day of testing.

The MWM task was used to evaluate spatial memory performance as described previously (Bermpohl et al., 2006; Mannix et al., 2011). The apparatus consisted of a circular black-colored water tank (120 cm in diameter and 50 cm high) filled with water to 29-cm depth with several highly visible cues located on the walls of each of the four quadrants. The water in the tank was colored by black non-toxic food pigment, and the temperature was maintained at 21–25°C. A clear plexiglass goal platform 5 cm in diameter was positioned 0.5 cm below the water’s surface approximately 15 cm from the southwest wall. Each mouse was subjected to a series of four to eight trials per day. For each trial, mice were randomized to one of four starting locations (north, south, east, or west) and placed in the pool facing the wall. Mice were given a maximum of 60 s to find the submerged platform. If the mouse failed to reach the platform by the allotted time, it was placed on the platform by the experimenter and allowed to remain there for 10 s. Mice were placed in a warming chamber for at least 4 min between trials. To control for possible differences in visual acuity or sensorimotor function between groups, two trials were performed using a visible platform raised 0.5 cm above the surface of the water. Performance in the MWM was quantitated by latency to find the platform. To minimize potential variability in performance due to daily environmental differences, mice were always tested concomitantly in motor and MWM tasks. The time to reach the visible platform was recorded and analyzed. Trajectories and latencies of trials were monitored and achieved using a video camera and analyzed with a tracking device and software (Chronotrack 3.0, San Diego Instruments).

### Administration of Propidium Iodide (PI) and Detection of PI-Positive Cells

PI (10 mg/ml; Sigma-Aldrich Corporation, St. Louis, MO, United States) was diluted in 0.9% NaCl and 0.4 mg/kg was administered 1 h before killing by intraperitoneal injection in a total volume of not more than 100 μl (Whalen et al., 2008; Park et al., 2012). Mice were killed at 24 h after brain injury, brain was frozen in nitrogen vapor, and cryostat brain sections (12 μm) were cut at 150- to 200-μm intervals from the anterior to posterior hippocampus (bregma –1.90 to –3.00). The cryostat sections were placed on poly-L-lysine slides and stored at –80°C. All cortical regions of the brain were chosen from 200 × cortical fields from within contused cortex. PI-positive cells were quantitated in the cortex and hippocampus in three brain sections separated by at least 150–200 μm (Whalen et al., 2008). For detection of PI-labeled cells, brain sections were fixed in 100% ethanol for 10 min at room temperature, coverslipped with Permount (Biomeda, Foster City, CA, United States), and photographed on a Nikon Eclipse T300 fluorescence microscope (Tokyo, Japan) using excitation/emission filters at 568/585 nm for PI.

### Double Immunofluorescence Staining

All sections were first blocked with 10% normal serum blocking solution species the same as the secondary antibody, containing 3% (w/v) BSA and 0.1% Triton X-100 and 0.05% Tween 20 2 h at room temperature in order to avoid unspecific staining. The sections were then incubated with different markers as follows: NeuN (neuron marker, 1:200; Abcam), glial fibrillary acidic protein (GFAP) (astrocyte marker, 1:200; Abcam), and CD11b (microcyte marker, 1:200; Abcam). Briefly, sections were incubated with both primary antibodies overnight at 4°C, followed by a mixture of fluorescein isothiocyanate-conjugated secondary antibodies for 2 h at 4°C. The stained sections were examined with a Leica spectral confocal microscope (Germany).

### Hemorrhagic Injury Volume Analysis

At 24 h after ICH, mice (*n* = 6/group) were killed and brains were harvested, fixed in 4% paraformaldehyde for 24 h, and cryoprotected in serial phosphate-buffered sucrose solutions (10, 20, and 30%) at 4°C, and then cut into 15-μm sections with a cryostat and 150 μm apart. Sections were stained with cresyl violet before being quantified for injury volume with Sigmacan Pro 5. Six coronal slices from different levels of the injured hemorrhagic area were summed, and the volumes in cubic millimeters were calculated by multiplying the thickness by the measured areas (Zhang et al., 2014). Six mice per group were analyzed by an observer blind to the experimental treatment.

### Western Blot Analysis

Mice were given an overdose of chloral hydrate and sacrificed at different time points postoperatively (*n* = 3 for each time point); tissues from the striatum of the injured hemisphere surrounding the wound (extending 2 mm to the incision) were for detecting the expression of protein by Western blotting technique, respectively. To prepare lysates, frozen brain tissue samples were minced with eye scissors in ice. The samples were then homogenized in lysis buffer [1% NP-40, 50 mmol/L tris, pH 7.5, 5 mmol/L EDTA, 1% SDS, 1% sodium deoxycholate, 1% Triton X-100, 1 mmol/L phenylmethylsulfonyl fluoride (PMSF), 10 μg/ml aprotinin, and 1 μg/ml leupeptin] and clarified by centrifugation for 20 min in a microcentrifuge at 4°C. After determination of its protein concentration with the Bradford assay (Bio-Rad), the resulting supernatant (20 μg of protein) was subjected to sodium dodecyl sulfate (SDS)-polyacrylamide gel electrophoresis (PAGE). The separated proteins were transferred to a polyvinylidene difluoride membrane (Millipore) by a transfer apparatus at 350 mA for 1.5 h. The membrane was then blocked with 5% non-fat milk and incubated with primary antibody against CBS (1:200; Santa Cruz Biotechnology, Santa Cruz, CA, United States), 3-mercaptopyruvate sulfurtransferase (MPST) (1:200; Santa Cruz Biotechnology, Santa Cruz, CA, United States), caspase 3 (1:500; Bioworld Technology, Minneapolis, MN, United States), Bcl-2 (1:1,000; Bioword Technology, Minneapolis, MN, United States), Beclin-1 (1:500; Santa Cruz Biotechnology, Santa Cruz, CA, United States), LC3B (1:3,000; Abcam, Cambridge, MA, United States), P62 (1:500; Santa Cruz Biotechnology, Santa Cruz, CA, United States), or actin (1:200; Santa Cruz Biotechnology, Santa Cruz, CA, United States). After incubating with an anti-rabbit or anti-mouse horseradish peroxidase-conjugated secondary antibody, protein was visualized using an enhanced chemiluminescence system (ECL, Pierce Company, United States).

### Statistics Analysis

All data were expressed as mean ± standard error of the mean (SEM). PI-positive cell count was analyzed by the rank sum test. Motor and MWM test data (hidden and visible platform acquisition latencies) were analyzed by two factor repeated-measures analysis of variance (ANOVA; for group and time) followed by *post hoc* Bonferroni’s test for multiple comparisons. Data on the probe trial were analyzed using one-way ANOVA analysis followed by *post hoc* Tukey’s test for multiple comparisons. Western blot data were carried out by one-way ANOVA with Dunnett’s *t* test. All analyses were performed with SPSS statistical package (version 13.0 for Windows, SPSS Inc., United States). For all comparisons, *P* < 0.05 was regarded as significant.

## Results

### H_2_S Attenuates ICH-Induced Brain Edema Through Endogenous H_2_S Synthesis Pathway in Mice

**FIGURE 1:**
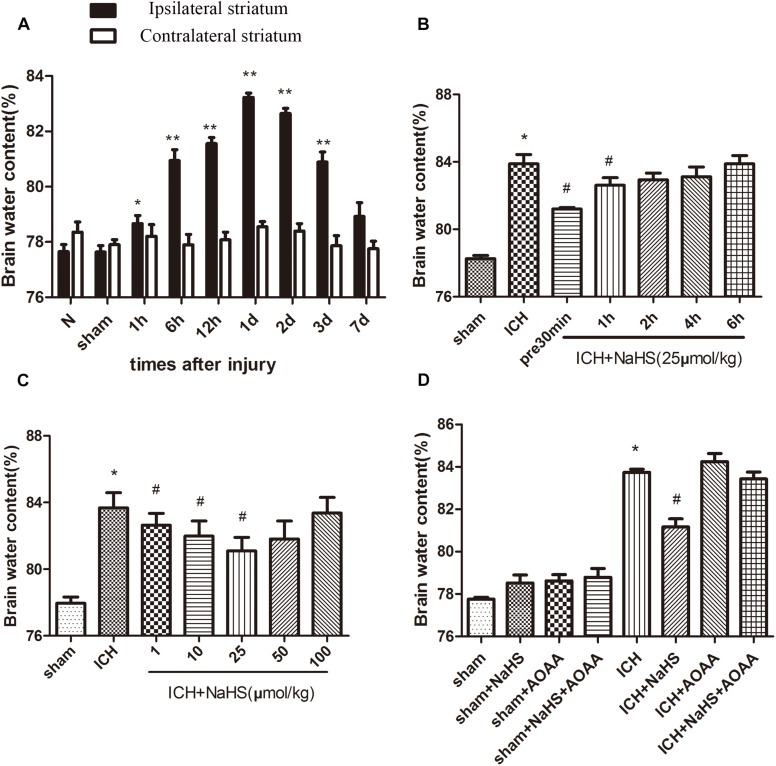
Treatment with H_2_S attenuated ICH-induced brain edema through the endogenous H_2_S synthesis pathway. **(A)** The water content of the injured hemisphere and contralateral hemisphere were measured from 1 h to 7 days after ICH. ^*^P < 0.05, ^∗∗^*P* < 0.01 vs. sham group. **(B)** Injected H_2_S (25 μmol/kg), at 30 min before or 1, 2, 4, or 6 h post-ICH, and the brain water content was detected at 1 day after ICH. ^*^P < 0.05 vs. Sham group. ^#^*P* < 0.05 vs. ICH group. **(C)** Pretreatment with H_2_S at 30 min before ICH with different dosages from 1 to 100 μmol/kg, and brain water content was measured at 1 d after ICH. ^*^P < 0.05 vs. sham group. #*P* < 0.05 vs. ICH group. **(D)** Pretreatment with H_2_S, AOAA, or combined treatment of H_2_S and AOAA before ICH, and then the brain water content was measured at 1 day after ICH. ^*^P < 0.05 vs. sham group. #*P* < 0.05 vs. ICH group. N indicates the normal group. Data are expressed as mean ± SEM (*n* = 6).

The percentage of brain water content significantly increased in the injured ipsilateral cortex after ICH. Brain water content in the injured hemisphere was increased starting at 1 h and peaking 1 day post-ICH compared with the contralateral hemisphere and sham group. Pretreatment with NaHS at doses of 1–25 μmol/kg 30 min before ICH can reduce brain water content ipsilateral to the injury, but NaHS with doses of 50 or 100 μmol/kg has no protective effect. Pretreatment with NaHS at doses of 25 μmol/kg 30 min before or 1 h after ICH attenuated ICH-induced brain edema (Figure 1). On the other hand, the combined treatment of H_2_S and AOAA exerted no greater inhibition of brain edema, suggesting that treatment with exogenous H_2_S can attenuate the development of brain water content induced by ICH through the endogenous H_2_S synthesis pathway.

### Exogenous H_2_S Induces CBS Expression and H_2_S Production Through the Endogenous H_2_S Synthesis Pathway

**FIGURE 2:**
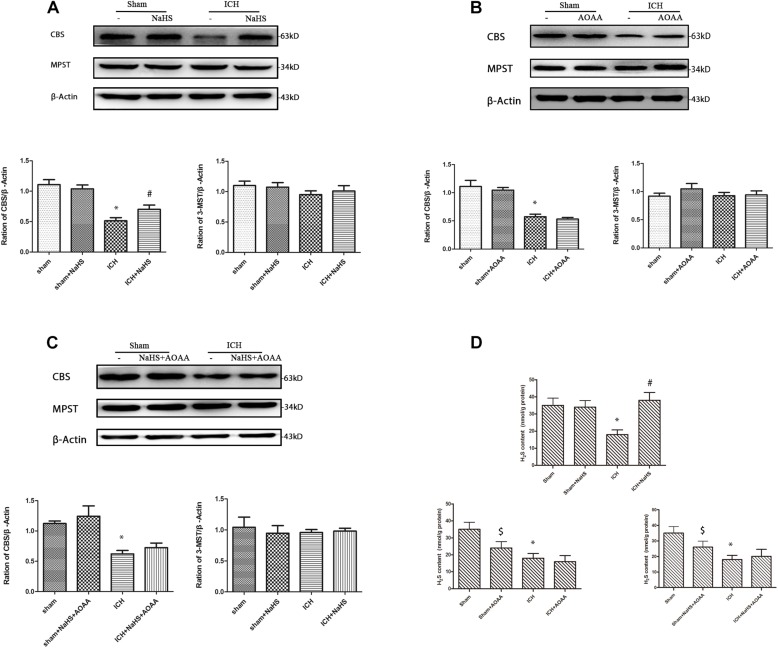
Western blot analysis showing the expression of CBS, MPST, and Glyceraldehyde 3-phosphate dehydrogenase (GAPDH) and H_2_S production in the striatum after ICH. Sample immunoblots probed for CBS, MPST, and GAPDH in the striatum are shown above. The bar chart below demonstrates the ratio of CBS relative to GAPDH and MPST relative to GAPDH for different treatment groups. **(A)** In the striatum, CBS protein decreased from post-injury and increased during pretreatment with NaHS. **(B)** There is no change on the expression of CBS and MPST when pretreated with the CBS inhibitor AOAA. **(C)** When treated with NaHS and AOAA, there are no changes in CBS and MPST expression compared with the ICH group. The data are mean ± SEM (*n* = 3, ^*^P < 0.05, significantly different from the sham groups). **(D)** Compared with the ICH group, NaHS supplementation induces H_2_S production. CBS inhibitor AOAA has no impact on CBS expression, but it reduces H_2_S production in the sham group. NaHS supplementation cannot induce H_2_S production in ICH mice when treated with AOAA. The data are mean ± SEM (*n* = 6, ^*^P < 0.05 vs. sham group. ^#^*P* < 0.05 vs. ICH group. ^[*dollar*]^*P* < 0.05 vs. sham group).

We found that ICH led to a significant decrease in expression of CBS and H_2_S production compared with the sham group. Compared with the ICH group, NaHS supplementation induces CBS expression and H_2_S production. CBS inhibitor AOAA has no impact on CBS expression, but it reduces H_2_S production in the sham group, suggesting that AOAA reduces the production of H_2_S by blocking the H_2_S-producing activity of CBS enzyme instead of decreasing CBS expression. When treated with NaHS and AOAA, there is no change in CBS expression and H_2_S production compared with the ICH group (Figure 2). NaHS supplementation cannot induce CBS expression and H_2_S production in ICH mice when treated with AOAA. We also detected the expression of other H_2_S-producing enzymes, such as 3-MST, and ICH led to no change in the expression of 3-MST compared with the sham group (Figure 2).

### H_2_S Attenuates Spatial Memory Impairment and Motor Deficits After ICH

In order to determine whether H_2_S could improve spatial memory acquisition and motor function, we performed the neurofunctional assessment using behavior tests.

**FIGURE 3:**
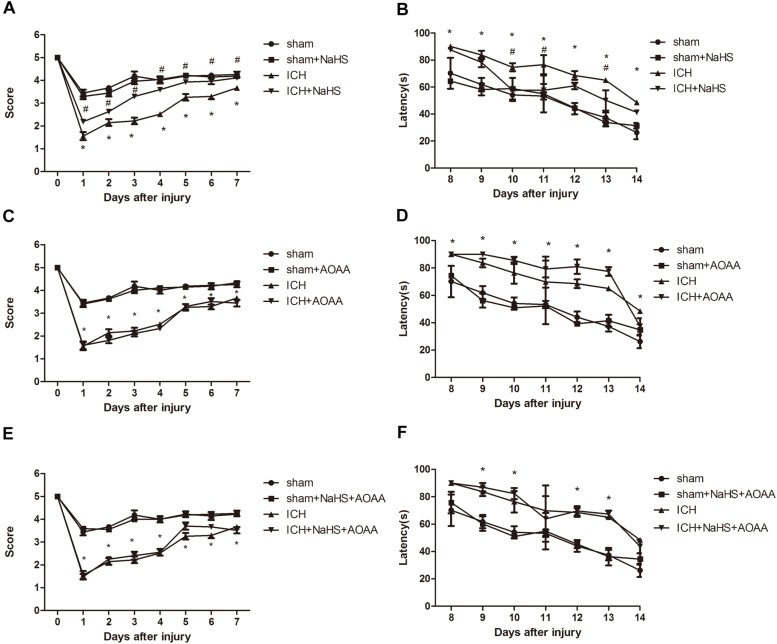
H_2_S improved the recovery of ICH-induced motor deficits and improved spatial memory acquisition through the endogenous H_2_S synthesis pathway. **(A,C,E)** Motor function was assessed by a wire grip test. No difference in baseline motor function before ICH was observed between groups of mice administered with H_2_S. Pretreatment with H_2_S speeds up the recovery of motor function deficits in mice compared to the ICH group. Pretreatment with AOAA alone could not improve the recovery of motor functional outcome post-ICH compared to the ICH group. Moreover, pretreatment with H_2_S could not improve the recovery of motor functional outcome on days 4–7 post-ICH when subjected to AOAA injection compared to the ICH group (^*^P < 0.05 vs. ICH group, *n* = 10/group). **(B,D,F)** Mice in the ICH group had significantly longer escape latency compared with sham group in the hidden platform task; however, ICH + NaHS group had significantly lower escape latency compared with the ICH group. Pretreatment with AOAA alone could not improve the recovery of cognitive functional outcome post-ICH compared to the ICH group. Moreover, pretreatment with NaHS could not reverse cognitive functional damage if the endogenous H_2_S production was inhibited by AOAA post-ICH compared to the ICH group. There was no significant difference in escape latency in all four groups in the visible platform task (^*^P < 0.05 vs. sham group; #*P* < 0.05 vs. ICH group, *n* = 10/group).

There was no significant differences between groups before ICH in the baseline of motor function. ICH elicited a significant decrease in the performance of motor function on days 1–7, which returned to preinjury levels on day 8 after ICH. Compared with the ICH group, there was a statistically significant improvement in motor recovery and functional outcome with 25 μmol/kg NaHS supplementation on days 4 –7 post-injury (Figure 3). We also found that pretreatment with AOAA alone could not improve the recovery of motor functional outcome post-ICH compared to the ICH group. Moreover, there was no significant improvement in motor recovery and functional outcome with 25 μmol/kg NaHS supplementation on days 4–7 post-injury when subjected to AOAA injection compared to the ICH group (Figure 3).

The MWM was used to evaluate spatial memory and was performed after day 8 when no significant difference in motor function was observed between different groups. In the visible or hidden platform test and probe trial, all groups of mice showed normal acquisition curves and selective quadrant search in MWM experiments before suffering ICH (Figure 3). Compared with the sham group, there is significant increase in the latencies to search the hidden platform on days 8–14 in the ICH group. Pretreatment with H_2_S displayed decreased latencies to find the hidden platform on days 10–13 compared with the ICH group, suggesting H_2_S could improve cognitive and functional recovery following ICH. Pretreatment with AOAA alone could not improve the recovery of cognitive functional outcome post-ICH compared to the ICH group. Moreover, pretreatment with H_2_S (25 μmol/kg) could not reverse cognitive functional damage if the endogenous H_2_S production was inhibited by AOAA after ICH compared to the ICH group (Figure 3). We performed visible platform testing on days 16 and 17 during the MWM test in order to avoid bias in the effect of visual acuity. In the visible platform test, no significant difference in latencies to find the visible platform was observed between groups on days 16 and 17. Compared with the sham group, there is a significant decrease in time in the target quadrant in the ICH group in the probe trial. Pretreatment with H_2_S displayed more time swimming in the target quadrant compared with the ICH group.

### H_2_S Attenuates Hemorrhage Volume and Permeability of Plasmalemma After ICH

**FIGURE 4:**
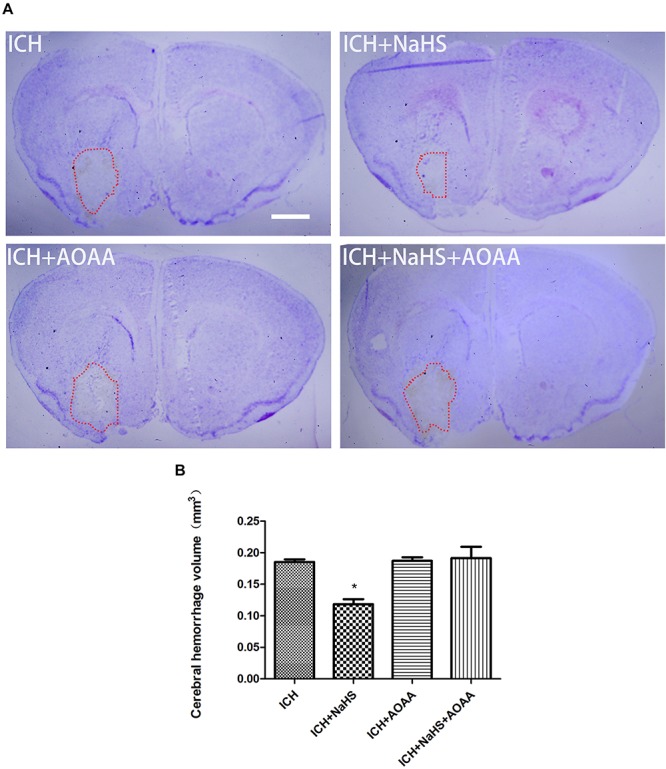
Pretreatment with H_2_S reduces injury volume at 24 h after ICH in the injured striatum. **(A)** Coronal sections were collected at 24 h after ICH and stained for cresyl violet. Pretreatment with H_2_S reduced injury volume after ICH in mice. However, pretreatment with H_2_S could not reduce injury volume if the endogenous H_2_S production was inhibited by AOAA post-ICH compared to the ICH group. **(B)** Quantification shows that brain injury volume was significantly smaller in the H_2_S-treated mice than in the ICH group mice (^*^P < 0.05 vs. ICH group, *n* = 6 in each group). Scale bars: 1 mm.

To determine the effect of H_2_S on hemorrhage volume caused by ICH, cresyl violet stain was used to identify the neuronal structure in the brain 24 h post-ICH. The results showed that pretreatment with H_2_S attenuated hemorrhage volume at 24 h following ICH (Figure 4). However, pretreatment with H_2_S could not reduce injury volume if the endogenous H_2_S production by AOAA after ICH was inhibited, compared to ICH group (Figure 4). No detectable hemorrhage was observed in the sham-operated mice.

**FIGURE 5:**
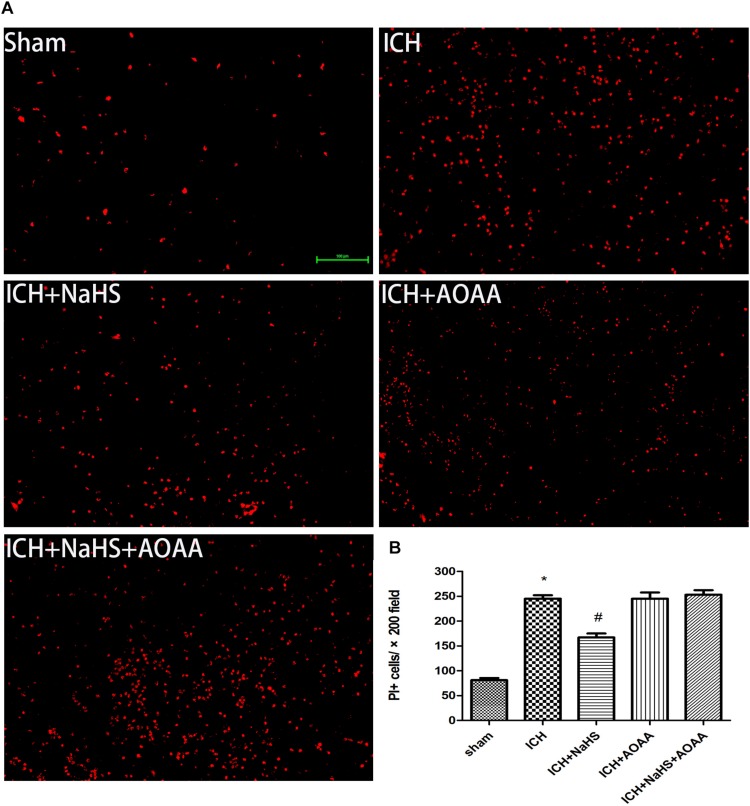
Pretreatment with H_2_S reduces PI-positive cells after ICH in the injured striatum. **(A)** Representative photomicrographs showed that pretreatment with H_2_S reduced numbers of PI-positive cells in the striatum brain regions after brain injury in the ICH + NaHS group compared to the ICH group. In contrast to injured mice, PI-positive cells were not detected in brain regions from the sham group or in the contralateral hemisphere of the injured mice. However, pretreatment with H_2_S could not decrease the number of PI-positive cells when inhibiting endogenous H_2_S production by AOAA compared to the ICH group. Original magnification × 200. **(B)** Quantitation of PI-positive cells in the injured striatum regions. ^*^P < 0.05 vs. sham group. ^#^*P* < 0.05 vs. ICH group (*n* = 10 per group).

**FIGURE 6:**
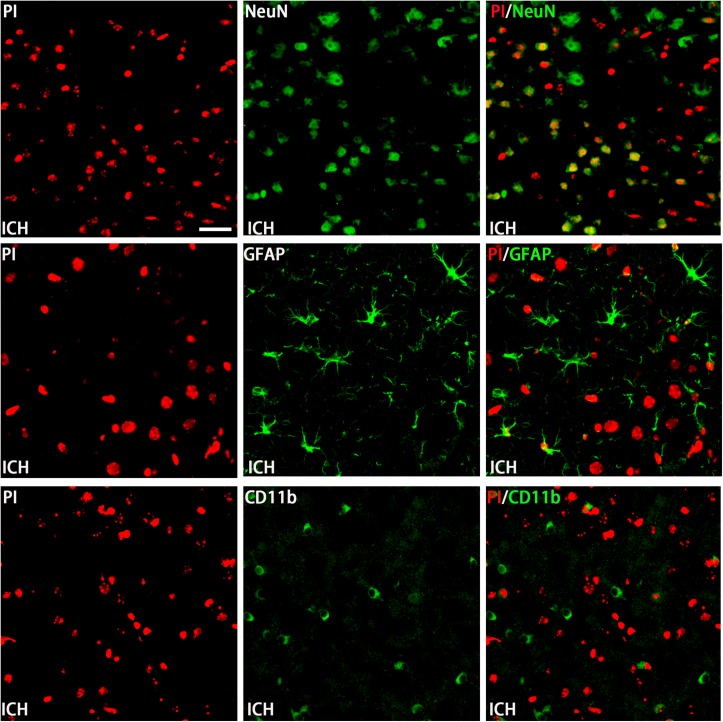
Double immunofluorescence staining for PI with NeuN, GFAP, and CD11b at the first day in the injured striatum after ICH. Coronal sections labeled with PI (red), NeuN (green), GFAP (green), and CD11b (green) and the yellow color visualized in the merged images represented colocalization of PI with NeuN (neuronal marker), PI with GFAP (astrocyte marker), and PI with CD11b (microglia marker). Double immunofluorescence staining revealed that at the first day post-injury, there was more colocalization between NeuN and PI. The GFAP-expressing astrocyte or CD11b-expressing microglia few partly showed as PI positive. Scale bars: 25 μm.

The permeability of plasmalemma was used for the detection of early stage of cell death, including autophagic cell death, apoptosis, and necrosis. To interpret cellular mechanisms of reduced hemorrhage volume, we investigated the effect of H_2_S on plasmalemma permeability in the striatum after injury using *in vivo* PI staining (Figure 5). Pretreatment with H_2_S reduced the numbers of PI-positive cells in the striatum at 6 h following ICH. There were no PI-positive cells to be detected in the striatum of the sham group mice and in the contralateral striatum region of the ICH mice. However, pretreatment with H_2_S could not decrease the number of PI-positive cells when inhibiting the endogenous H_2_S production by AOAA after ICH compared to ICH group (Figure 5). In order to distinguish the cell type of the PI-positive cells, we used double immunofluorescence staining for PI with NeuN (neuronal marker), PI with GFAP (astrocyte marker), and PI with CD11b (microglia marker). Double immunofluorescence staining revealed that there was more colocalization between NeuN and PI (Figure 6). The GFAP-expressing astrocyte or CD11b-expressing microglia partly showed as PI positive. These results suggested that ICH induced neuronal death in the striatum following ICH, and pretreatment with H_2_S may reduce neuronal death in the striatum following ICH.

### H_2_S Inhibits the Activation of Caspase3 and the Reduction in Bcl-2

**FIGURE 7:**
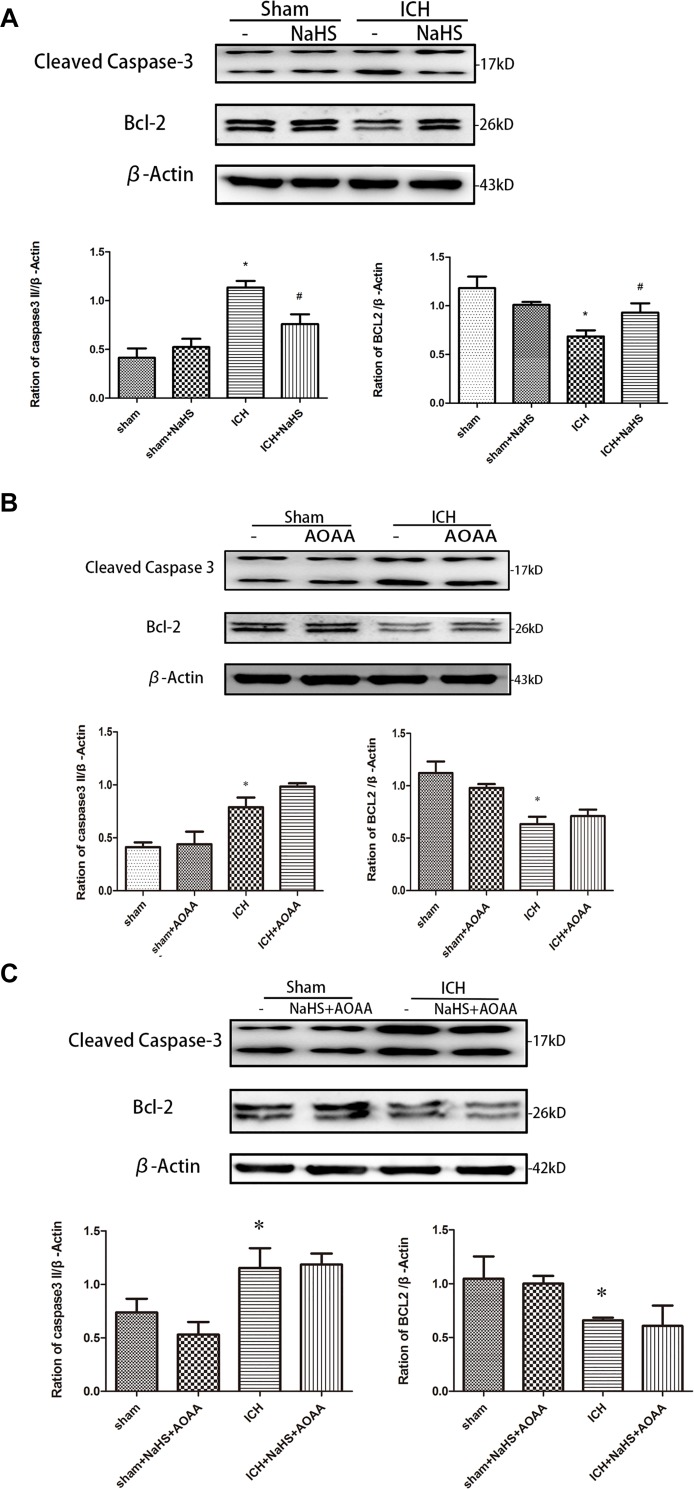
ICH-induced cleavage of caspase 3 and Bcl-2 decline were reversed by H_2_S pretreatment. Sample immunoblots probed for caspase 3, Bcl-2, and actin are shown above. The *bar chart* below demonstrates caspase 3 and Bcl-2 relative to actin. **(A)** ICH-induced upregulation of cleaved caspase 3 and downregulation of Bcl-2 were inhibited by H_2_S in the striatum. **(B,C)** However, pretreatment with H_2_S could not suppress ICH-induced upregulation of cleaved caspase 3 and downregulation of Bcl-2 if endogenous H_2_S production by AOAA post-ICH was inhibited compared to the ICH group. Optical densities of the protein bands were quantitatively analyzed with Sigma Scan Pro 5 and normalized with loading control actin. The data are means ± SEM (*n* = 3, ^*^P < 0.05, ICH group vs. sham group; #*P* < 0.05, ICH + NaHS group vs. ICH group at the same time point).

Because loss of plasmalemma integrity is in the early stage of cell death, we investigated whether pretreatment with H_2_S could inhibit the activation of cell apoptosis in the striatum at 24 h after ICH by detecting the expression of caspase 3 and Bcl-2 (Figure 7). There was a significant increase in the expression of cleaved caspase 3 and a decrease in the expression of Bcl-2 in the injured striatum at 24 h following ICH. However, pretreatment with H_2_S inhibited the activation of caspase 3 and the reduction in Bcl-2, suggesting pretreatment with H_2_S could inhibit the activation of cell apoptosis in the injured striatum. However, pretreatment with H_2_S could not suppress cell apoptosis induced by ICH if the endogenous H_2_S production was inhibited by AOAA post-ICH compared to the ICH group (Figure 7).

### H_2_S Reversed the Upregulation of LC3 and Beclin-1 and the Downregulation of p62 Induced by ICH

**FIGURE 8:**
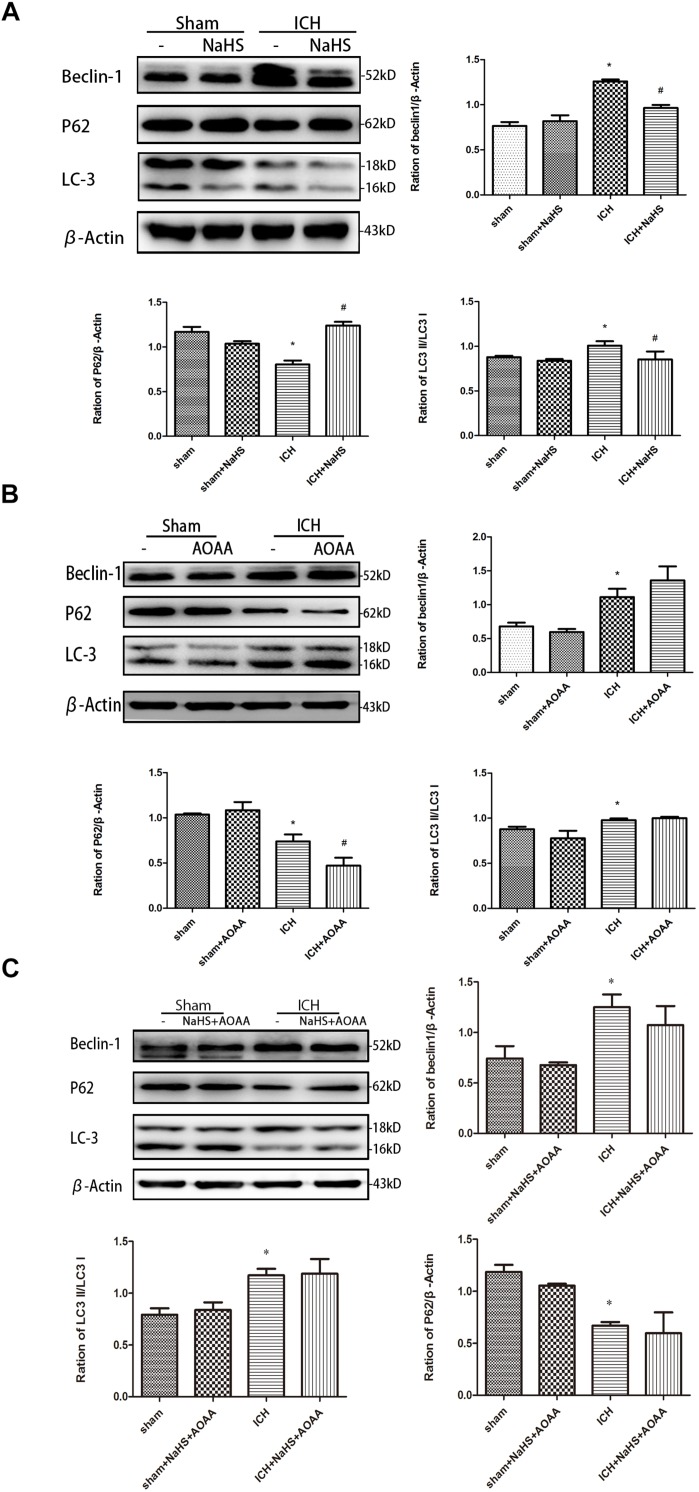
H_2_S pretreatment reversed ICH-induced LC3II, P62, and Beclin-1 increase. Sample immunoblots probed for LC3II, P62, Beclin-1, and actin are shown above. The *bar chart* below demonstrates the ratio of LC3II, P62, and Beclin-1 relative to actin. **(A)** ICH-induced upregulation of LC3II and Beclin-1 and downregulation of P62 were inhibited by H_2_S in the striatum. **(B,C)** However, pretreatment with H_2_S could not suppress ICH-induced upregulation of LC3II and Beclin-1 and downregulation of P62 if endogenous H_2_S production by AOAA post-ICH was inhibited compared to the ICH group. Optical densities of the protein bands were quantitatively analyzed with Sigma Scan Pro 5 and normalized with loading control GAPDH. The data are means ± SEM (*n* = 3, ^*^P < 0.05, ICH group vs. sham group; #*P* < 0.05, ICH + NaHS group vs. ICH group at the same time point).

We detected the expression of LC3II, p62, and Beclin-1 by Western blotting assay in order to determine whether pretreatment with H_2_S could inhibit the activation of autophagy (Figure 8). The expressions of LC3II and Beclin-1 protein are the specific markers of autophagy, reflecting the progress of autophagy such as autophagosome formation and maturity. p62 protein serves as a useful marker for monitoring the autophagic flux, which reflects autophagic clearance of protein aggregate (Ichimura et al., 2008). Therefore, we detected the expression of LC3II and Beclin-1 and then found that there was a significant increase in the expression of LC3II and Beclin-1 in the striatum following ICH. However, pretreatment with H_2_S inhibited the upregulation of LC3II and Beclin-1, suggesting H_2_S could inhibit the activation of autophagy in the injured striatum (Clark et al., 2008). There was a significant decrease in the expression of p62 in the striatum following ICH, and pretreatment with H_2_S restored p62 protein level to the preinjury range. Furthermore, we detected the expression of LC3II, p62, and Beclin-1 in the ICH group when pretreated with AOAA or pretreated with NaHS and AOAA, and then we found that H_2_S synthesis inhibitor AOAA inhibited the protective effects of H_2_S (Figure 8).

## Discussion

Hemorrhagic stroke (ICH) is caused by sudden blood vessel rupture, which accounts for the remaining 10%–15% of strokes. It is a pathological accumulation of blood in the brain parenchyma and is associated with high mortality and morbidity (Bobinger et al., 2018). Although we have much knowledge about the molecular pathophysiology of ICH on the cellular level, we have not yet developed a safe and effective neuroprotective drug to prevent cell damage caused by stroke. An ideal neuroprotectant would be high permeability to the blood–brain barrier (BBB), which played a protective role during the processes of nerve injury and had considerable efficacy and safety. Over the last few decades, there is significant progress achieved in delineating the therapeutic potentials and molecular mechanisms underlying the actions of H_2_S on brain diseases (Paul and Snyder, 2018). Our previous study demonstrated that H_2_S provided neuroprotection in TBI *via* inhibition of autophagy and apoptosis, but the molecular mechanisms involved in the cytoprotective effect of H_2_S after brain injury have not been investigated (Zhang et al., 2014). A gap still exists between neuroprotection effect of exogenous H_2_S and the molecular mechanism of endogenous H_2_S on brain injury. Moreover, no clear mechanisms of exogenous H_2_S modulating the endogenous H_2_S pathway were addressed, and biological effects of endogenous H_2_S synthase inhibitors were not investigated after ICH. In order for us to develop therapies designed to stimulate this repair, we explored potential neuroprotective mechanisms of H_2_S against ICH-induced injury.

In this study, we demonstrated for the first time that the protective effect of exogenous H_2_S against ICH may be not a direct action but an indirect effect through inducing endogenous H_2_S metabolism responses. Another important observation we made is that AOAA, an inhibitor of CBS, significantly reduced production of endogenous H_2_S, but AOAA has no impact on the expression of CBS, suggesting that AOAA reduced endogenous H_2_S production through inhibiting the enzyme activity of CBS rather than modulating the expression of the CBS protein level. We also distinguished cell types of PI-positive cells in an *in vivo* mice ICH model using double immunofluorescence staining PI with NeuN (neuronal marker), PI with GFAP (astrocyte marker), and PI with CD11b (microglia marker). Double immunofluorescence staining revealed that there was more colocalization between NeuN and PI. The GFAP-expressing astrocyte or CD11b-expressing microglia partly showed as PI positive. It suggested that ICH-induced neuronal death in the striatum following ICH and pretreatment with H_2_S may reduce neuronal death in the striatum following ICH. We also demonstrated that endogenous H_2_S production was significantly decreased in the striatum after ICH as a result of a decrease in CBS protein levels. H_2_S pretreatment also reversed ICH-induced apoptosis and autophagy in injured striatum post-ICH. In addition, we demonstrated that pretreatment with H_2_S attenuated brain water content and exhibited improved performance in sensory motor and cognitive function following ICH. Cerebral edema, as a marker of secondary injury after ICH, may result in poor prognosis in patients with ICH. There is increasing interest in the mechanisms of secondary brain injury as a therapeutic target. It is therefore of pressing concern to identify cerebral edema as a proof-of-concept alternative to evaluate the feasibility, acceptability, and safety of different types of therapy for ICH (Selim and Norton, 2018). Therefore, cerebral edema is used as the evaluation standard to determine the therapeutic efficiency of H_2_S on brain damage induced by ICH. In this study, we demonstrated that brain water content in the injured hemisphere was increased starting at 1 h and peaked 1 day post-ICH. Pretreatment with NaHS at doses of 1–25 μmol/kg 30 min before ICH can reduce ipsilateral brain water content after injury, but NaHS with doses of 50 or 100 μmol/kg has no protective effect. The most effective concentration of H_2_S against brain edema after ICH is 25 μmol/kg, and the time window for H_2_S treatment in ICH is 30 min prior to hemorrhage onset. We will use the same drug concentration for subsequent studies to explore the efficacy of H_2_S in attenuating brain injury following ICH.

There are three enzymes for the generation of endogenous H_2_S in the mammalian cell: CBS, MPST, and cystathionine-γ-lyase (CSE) (Zhang J.Y. et al., 2017). Previous studies have shown that CBS and MPST are major H_2_S-producing enzymes in the brain (Ishigami et al., 2009; Zhang M. et al., 2017). Our results showed that there was a significant decrease in the expression of CBS following ICH, but statistically significant differences in the expression of MPST between the two groups were not detected, suggesting that CBS is the major contributor to H_2_S production in the brain following ICH. Although the molecular mechanism that CBS expression was downregulated following ICH accompanied with unchanged MPST is not clear, we provide further evidence and new results showing that the downregulation of CBS expression plays a vital role in neurologic impairment after injury. NaHS, a classical exogenous H_2_S donor, significantly increases the expression of CBS and production of endogenous H_2_S after ICH-induced injury. Another important observation we made is that AOAA, an inhibitor of CBS, significantly reduced production of endogenous H_2_S after ICH, but AOAA have no impact on the expression of CBS, suggesting that AOAA reduced endogenous H_2_S production through inhibiting the enzyme activity of CBS rather than modulating the expression of CBS protein level. Moreover, NaHS supplementation cannot induce CBS expression and H_2_S production in ICH mice when treated with AOAA. These data suggest that NaHS as an exogenous H_2_S donor mimics the effect of endogenous H_2_S after ICH *via* modulating CBS enzyme activity, further indicating that impaired CBS–H_2_S signaling axis contributes to brain damage in a mouse model of ICH. AOAA did not produce any significant effect such as cerebral edema in healthy control mice. This also suggested that the role of CBS may not be as important in health as in the disease state.

A majority of patients who are ICH survivors have some neurocognitive impairments, including complex cognitive abilities such as memory and executive function (Garcia et al., 2013; Salihu et al., 2016). In order to determine whether H_2_S could improve spatial memory acquisition and motor function, we performed a neurofunctional assessment using behavior tests. Pretreatment with H_2_S displayed significant improvement in motor recovery and cognitive functional outcome following ICH. However, if endogenous H_2_S production by AOAA was inhibited, pretreatment with NaHS could not reverse cognitive functional damage compared to the ICH group. These results suggested that exogenous H_2_S displayed significant improvement in motor recovery and cognitive functional outcome following ICH through the endogenous H_2_S synthesis pathway. The neuronal cell loss caused by ICH-induced cell death is responsible for significant patient disability, mortality, and morbidity after ICH (Bobinger et al., 2018). Numerous factors that trigger post-ICH pathophysiological pathways lead to cell death in the perihematomal and remote brain regions (Zille et al., 2017). *In vivo* PI was used to assess plasmalemma damage and cell death after ICH in mice (Zhu et al., 2012; Wang et al., 2013). Double immunofluorescence staining revealed that there was more colocalization between NeuN and PI after ICH. The GFAP-expressing astrocyte or CD11b-expressing microglia partly showed as PI positive. It suggested that ICH induced neuronal death in the striatum. Our results demonstrated pretreatment with H_2_S decreased the number of PI-positive cells in the injured striatum in mice after ICH, suggesting necrosis contributes to cell demise after ICH. While necrosis is considered non-programed cell death, cell death can occur in a programed manner. One example of programed cell death is apoptosis. The morphological hallmarks of apoptosis include cell shrinkage and membrane blebbing with no organelle changes. Caspase 3, a member of the caspase family, has been identified as a key mediator of neuronal programed cell death. It can be activated by two main apoptotic pathways: extrinsic death receptor pathway and intrinsic mitochondrial pathway (Gong et al., 2001; Qureshi et al., 2003). We found that pretreatment with NaHS inhibited the activation of caspase 3 and the reduction in Bcl-2 in the striatum, suggesting that H_2_S suppressed the activation of apoptosis in the striatum following ICH. Our previous study showed that autophagic cell death was involved in the pathology of ICH, and inhibition of autophagy as a therapeutic strategy provided neuroprotective effect in the ICH model (Shen et al., 2016; Gao et al., 2017). We detected the expression of LC3II, p62, and Beclin-1 by Western blotting assay in order to determine whether pretreatment with H_2_S could inhibit the activation of autophagy. The results showed that pretreatment with H_2_S inhibited the upregulation of LC3II and Beclin-1 and the downregulation of p62, suggesting H_2_S may provide a neuroprotective effect through inhibiting the autophagic cell death pathway. In addition, we detected apoptosis and autophagy when pretreated with NaHS and AOAA, and then we found that H_2_S synthesis inhibitor AOAA inhibited the protective effects of H_2_S through the apoptosis and autophagic cell death pathway.

**FIGURE 9:**
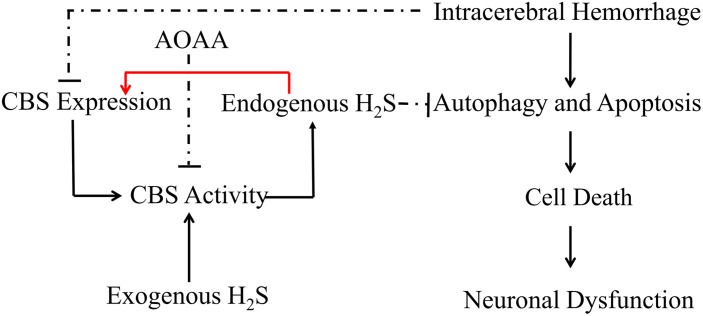
Hypothetical scheme outlining the mechanisms involved in the protective effects of H_2_S on cell injury induced by ICH. Intracerebral hemorrhage inhibited the expression of CBS and decreased the production of H_2_S in the mice model. H_2_S ameliorates brain edema and behavioral symptoms in ICH models. H_2_S may serve as a neuroprotectant to treat ICH-induced brain injury *via* antiapoptosis and suppression of excessive activation of autophagy. The protective effect of exogenous H_2_S against ICH may not be a direct action but an indirect effect through inducing endogenous H_2_S metabolism responses. Solid lines indicate activation, and dotted lines indicate inhibition.

In conclusion, the present study demonstrates that pretreatment with H_2_S attenuates ICH-induced brain water content and improves behavioral outcomes in a mice model of ICH. H_2_S exhibits neuroprotective effects through suppressing apoptosis and autophagic cell death following ICH and, therefore, may be recognized as a neuroprotectant in clinical therapy to limit neurological damage following hemorrhagic insults. The protective effect of exogenous H_2_S against ICH may be not a direct action but an indirect effect through inducing endogenous H_2_S metabolism responses (Figure 9).

## Ethics Statement

All the animal procedures were approved by the Institutional Animal Use and Care Committee at Soochow University and conducted in accordance with the guidelines of Animal Use and Care of the National Institutes of Health (NIH) and the ARRIVE (Animal Research: Reporting *In Vivo* Experiments). All efforts were made to minimize the numbers of animals used and ensure minimal suffering. In all experiments, data were obtained by investigators blinded to study group.

## Author Contributions

MZ, HS, and LT designed the study. HS, PC, JQ, YC, GC, and MZ performed the experiments and analyzed the data. HS, PC, CG, HW, CL, TW, and MZ interpreted the data for the work. MZ, HS, XC, and LT wrote the manuscript.

## Conflict of Interest Statement

The authors declare that the research was conducted in the absence of any commercial or financial relationships that could be construed as a potential conflict of interest.
